# A novel approach for quantitatively distinguishing between anthropogenic and natural effects on paleovegetation

**DOI:** 10.1093/pnasnexus/pgae135

**Published:** 2024-03-29

**Authors:** Baoshuo Fan, Houyuan Lu, Yuecong Li, Caiming Shen, Qinghai Xu, Jianping Zhang, Xiujia Huan, Yonglei Wang, Ningyuan Wang, Deke Xu, Yajie Dong, Anning Cui, Naiqin Wu

**Affiliations:** Hebei Key Laboratory of Environmental Change and Ecological Construction, College of Geographical Sciences, Hebei Normal University, Shijiazhuang 050024, PR China; Key Laboratory of Cenozoic Geology and Environment, Institute of Geology and Geophysics, Chinese Academy of Sciences, Beijing 100029, PR China; Key Laboratory of Cenozoic Geology and Environment, Institute of Geology and Geophysics, Chinese Academy of Sciences, Beijing 100029, PR China; University of Chinese Academy of Sciences, Beijing 100049, PR China; Hebei Key Laboratory of Environmental Change and Ecological Construction, College of Geographical Sciences, Hebei Normal University, Shijiazhuang 050024, PR China; Yunnan Key Laboratory of Plateau Geographical Processes and Environmental Changes, Faculty of Geography, Yunnan Normal University, Kunming 650500, PR China; Hebei Key Laboratory of Environmental Change and Ecological Construction, College of Geographical Sciences, Hebei Normal University, Shijiazhuang 050024, PR China; Key Laboratory of Cenozoic Geology and Environment, Institute of Geology and Geophysics, Chinese Academy of Sciences, Beijing 100029, PR China; Key Laboratory of Cenozoic Geology and Environment, Institute of Geology and Geophysics, Chinese Academy of Sciences, Beijing 100029, PR China; Shandong Provincial Key Laboratory of Soil Conservation and Environmental Protection, School of Resources and Environment, Linyi University, Linyi 276000, PR China; Zhejiang Provincial Institute of Relics and Archaeology, Hangzhou 310014, PR China; Zhejiang Provincial Institute of Relics and Archaeology, Hangzhou 310014, PR China; Key Laboratory of Cenozoic Geology and Environment, Institute of Geology and Geophysics, Chinese Academy of Sciences, Beijing 100029, PR China; Key Laboratory of Cenozoic Geology and Environment, Institute of Geology and Geophysics, Chinese Academy of Sciences, Beijing 100029, PR China; Key Laboratory of Cenozoic Geology and Environment, Institute of Geology and Geophysics, Chinese Academy of Sciences, Beijing 100029, PR China; Key Laboratory of Cenozoic Geology and Environment, Institute of Geology and Geophysics, Chinese Academy of Sciences, Beijing 100029, PR China

**Keywords:** pollen, paleovegetation, human activity, error inflection point-discriminant technique, Liangzhu

## Abstract

How to distinguish and quantify past human impacts on vegetation is a significant challenge in paleoecology. Here, we propose a novel method, the error inflection point-discriminant technique. It finds out the inflection points (IPs) of the regression errors of pollen–climate transfer functions using modern pollen spectra from vegetation with different values of the Human Influence Index (HII), which represent the HII threshold values of native/secondary and secondary/artificial vegetation systems. Our results show that the HII value at the native/secondary vegetation IPs is approximately 22 and globally uniform, whereas it varies regionally for the secondary/artificial vegetation IPs. In a case study of the Liangzhu archaeological site in the lower Yangtze River, discriminant functions for pollen spectra from three vegetation types and pollen–climate transfer functions of the native vegetation were established to reconstruct paleovegetation and paleoclimate over the past 6,600 years. Our study demonstrates this method's feasibility for quantitatively distinguishing human impacts on paleovegetation and assessing quantitative paleoclimate reconstructions using pollen data.

**Significance Statement**

Human activities, primarily deforestation and agriculture, have significantly influenced natural vegetation dynamics, especially during the middle and late Holocene. However, there are significant challenges in distinguishing and quantifying the effects of human activities on natural vegetation, which hinder the quantitative reconstruction of paleovegetation and paleoclimate. We present a novel method, the error inflection point-discriminant technique, which identifies the inflection points at which human activities significantly disturbed the natural vegetation. We found that a Human Influence Index value of 22 is the critical threshold at which human activities impacted the natural vegetation, and this value is globally consistent.

## Introduction

The relationship between past human activities and vegetation ecosystems is a critical issue in Quaternary ecosystem science (1). Past human activities, such as deforestation and agricultural practices, especially during the middle and late Holocene, have significantly influenced natural vegetation dynamics and thus the distribution patterns of modern and fossil pollen as proxies of modern and past vegetation (2–4). This process introduces biases in the quantitative reconstructions of past regional vegetation and climate using modern and fossil pollen data (5, 6). Distinguishing and quantifying the effects of past human activities on natural vegetation and their implications for paleovegetation and paleoclimate reconstructions remain a challenge for the paleoecological community (7, 8).

In recent decades, pollen data were extensively used to reconstruct Holocene paleovegetation and paleoclimate, and these studies tended to focus on three aspects: (i) quantitative reconstructions of paleovegetation and paleoclimate using fossil pollen records from lakes, peatlands, and loess in regions with minimal human impacts (9–11); (ii) archaeobotanical and paleoecological interpretation of fossil pollen records from archaeological sites or sedimentary sections impacted significantly by human activities (12–14), in which human utilizations of plant species and the effects of human activities on vegetation rather than changes in natural vegetation and climate were addressed (15–17); (iii) vegetational and climatic interpretation of fossil pollen records from regions where the vegetations experienced less human impacts in the early Holocene and different degrees of human activity influences in the middle and late Holocene, in which natural and human-affected vegetation changes were concerned (18, 19). All three aspects involve the reconstruction of paleovegetation and paleoclimate, and raise the question of whether modern and fossil pollen spectra are derived from natural vegetation or from vegetation affected by human activities, particularly the third aspect, which involves the challenging task of determining the stages of human-induced vegetation changes (17, 19–21).

This issue mainly involves two aspects: (i) how to eliminate pollen samples of human-affected vegetations from modern pollen training sets used to establish transfer functions (22). In the establishment of transfer functions, this step is generally completed qualitatively and artificially, and it lacks quantified and repeatable selection criteria (23); and it was ignored in some studies (8, 24). (ii) How to identify whether fossil pollen spectra are from past natural or human-affected vegetations (25, 26). Some studies used anthropogenic pollen indicators such as Poaceae pollen of cereals (≥35 or 38 μm) and Brassicaceae pollen, as well as the increased pollen presence of pioneer plants and the decreased pollen presence of arboreal plants as means of determining past human-affected vegetation (27–31). Again, however, no quantitative and repeatable criteria are available for identifying fossil pollen spectra from past human-affected vegetations and thus excluding them for the quantitative reconstruction of paleovegetation and paleoclimate (28, 30). It remains unclear to what extent human activities in distinct ecological regions of the world, both modern and past, have influenced modern and fossil pollen spectra, thereby impacting the reconstruction of paleovegetation and paleoclimate (2, 24, 32).

Therefore, it is essential to elucidate the extent of human impacts on the quantitative reconstruction of paleovegetation for the dependable quantitative reconstruction and prediction of past and future climate change using pollen data (33, 34). Theoretically, the coevolution of plants and animals in natural ecosystems as a natural process is driven by climatic and environmental changes (35, 36). The influence of animals (including early humans) on vegetation has been a longstanding presence within this natural process (18, 37). How can we determine whether the ecological characteristics of natural vegetation—including composition, abundance, structure, and life form—have deviated from this natural process due to human activities? Essentially, it is necessary to determine whether or not the ecological characteristics of natural vegetation are determined primarily by natural hydrothermal gradients (38, 39).

Modern pollen training sets used for deriving pollen–climate transfer functions consist of modern pollen samples from native and nonnative vegetation systems (see Methods) (40). The native vegetation is mainly controlled by temperature and precipitation and is minimally affected by human activities (19). The nonnative vegetation involves vegetation in urban and rural regions, agricultural fields, and other regions affected by human activities (40). The varying degrees of human influence in these cases can be defined explicitly as Human Influence Index (HII, values ranging from 0 to 64, with higher scores indicating greater human influence, see Methods), an index defined by the Wildlife Conservation Society and the Columbia University Center for International Earth Science Information Network (41). Modern pollen–climate transfer functions are developed under the assumption that the spatial distribution of modern natural vegetation is controlled by temperature and precipitation gradients (19). The greater the human impact on vegetation communities, the more these communities deviate from the natural drivers, i.e. temperature and precipitation gradients, leading to larger validation regression errors and smaller correlation coefficients in the established pollen–temperature/precipitation transfer functions (23). Whereas if the modern pollen training sets used to develop transfer functions are from natural vegetation systems, the resulting transfer functions have relatively small validation regression errors and larger correlation coefficients (42).

Theoretically, the validation errors of transfer functions established by modern pollen training sets from native and nonnative vegetation systems exhibit a random distribution, and samples from different systems follow distinct probability distributions (43, 44). The probability cumulative curve of error probabilities with changing HII values will show a significant turning point called the inflection point (IP) at a specific HII value. This IP at a specific HII value (the HII threshold value) serves as a definitive marker that differentiates between native and nonnative vegetation systems (see Methods). Conceptionally, it is similar to the IPs on the probability cumulative curve of grain-size components for distinguishing different dynamic systems (e.g. rolling, saltation, and suspension) in grain-size analysis (45). Therefore, we can use IPs: (i) to establish discriminant functions using modern pollen training sets from either native or nonnative vegetation systems, and apply them to fossil pollen spectra to determine their sources (i.e. native vs. nonnative vegetation systems) and (ii) to establish modern-climate transfer functions using modern pollen training sets from native vegetation system, and apply them to fossil pollen spectra from native achieves to reconstruct paleotemperature and paleoprecipitation.

**Fig. 1.**
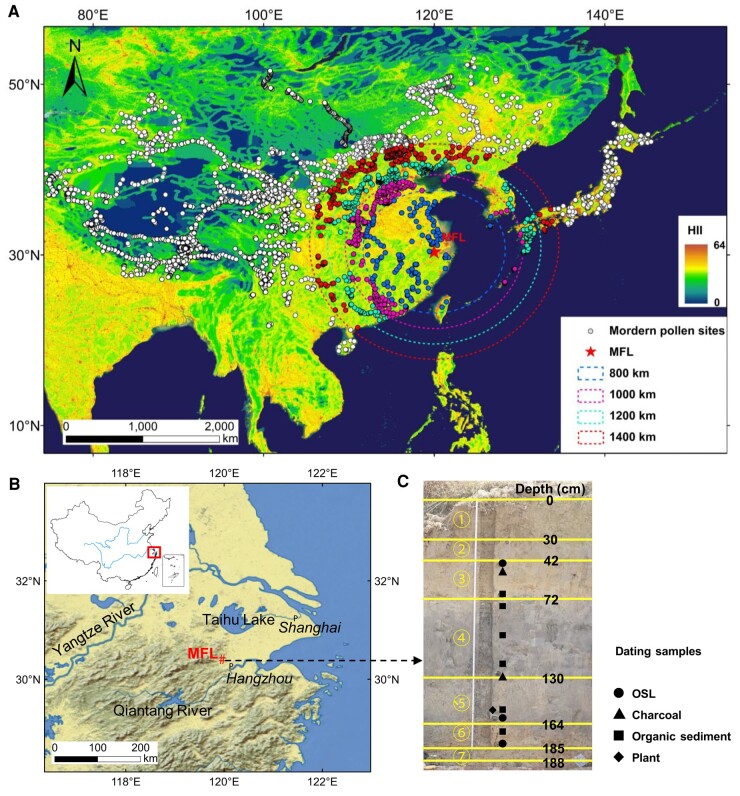
Locations of modern and fossil pollen samples and the stratigraphy of the studied section. A) Distribution of 6,053 modern pollen samples in East Asia (dots) (40). The four circles outlined represent distances of 800, 1,000, 1,200, and 1,400 km from the Liangzhu site. The HII values range from 0 to 64, with higher values indicating stronger human influence (41). B) Geographical location of the MFL profile at the Liangzhu site (46) (Figs. S1 and S2). C) Distribution of the MFL stratigraphic section and dating samples (Fig. S3). The MAT/°C of the MFL is ∼16°C, and the MAP/mm is ∼1,300 mm (47) (SI Appendix, Text C).

Here, we present a novel approach, termed the error inflection point-discriminant technique (EIPDT). Its objective is to quantitatively reconstruct paleovegetation and paleoclimate on the basis of identifying IPs in different vegetation regions worldwide and excluding human influences on vegetation as much as possible. In a case study, the EIPDT was applied to a 6,600-yr fossil pollen record from the Mifenglong (MFL) paleolake (119.983°E, 30.397°N, 30 m a.s.l.) at the Liangzhu archaeological site within the lower Yangtze River, China (46, 47) (Fig. 1; SI Appendix, Text A–C). The results enable us to quantitatively distinguish between anthropogenic and natural effects on paleovegetation.

## Results and discussion

### The IPs of native and nonnative vegetation systems in the middle and lower Yangtze River over eastern China

**Fig. 2.**
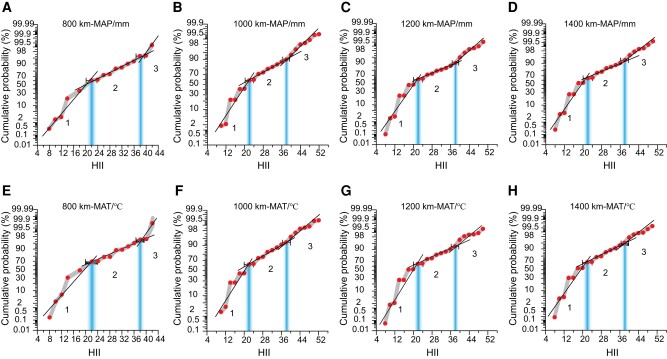
Cumulative probability curves of the regression errors of MAP/mm and MAT/°C in modern pollen–climate transfer functions (WA-PLS) over eastern China. Modern pollen samples derived from within the ranges of 800 km (245 samples) A, E), 1,000 km (681 samples) B, F), 1,200 km (1,155 samples) C, G), and 1,400 km (1,777 samples) D, H) from the MFL profile at the Liangzhu site. The red dots represent the cumulative regression error probabilities for different HII values. The gray shaded area represents the error range. The trend lines of the three scatter plots, characterized by different slopes, indicate distinct vegetation systems. The point of intersection between two linear segments marks the boundary between the two systems. The vertical axis uses a probability percentage scale. Probability percentage coordinates are nonequidistant coordinates with 50% as the center of symmetry and are plotted assuming a normal distribution (see Methods, Data S1).

We used the EIPDT (see Methods) to analyze the modern pollen data within circular areas with radii of 800, 1,000, 1,200, and 1,400 km from the center of the Liangzhu site in the lower Yangtze River. The regression errors of modern pollen–climate transfer functions within these four ranges were estimated. For the pollen–precipitation/temperature transfer functions of each radius, the results reveal three linear segments on the cumulative probability curve of the regression error with changing HII (Fig. 2). These three linear segments are defined by the two HII values of 22 ± 1.9 and 38 ± 1.2 as IPs. We label linear segments with HII ≤ 22, 22–38, and ≥ 38 as linear segments 1, 2, and 3, respectively.

According to the original vegetation records of the modern pollen samples (40), more than 92.7% of the samples with HII values ≤ 22 are distributed within natural forests, grasslands, and other naturally driven vegetation types, indicating that modern pollen samples with HII values ≤ 22 are mainly from native vegetation controlled by natural hydrothermal systems. Therefore, linear segment 1 represents a native vegetation system (Fig. 2). Modern pollen samples with HII values of 22–38 are mainly distributed within secondary vegetation (∼54.5%) and farmland (∼45.3%) surrounded by secondary vegetation, implying that those samples are from secondary vegetation affected by both natural and anthropogenic forcing. Linear segment 2 thus represents a secondary vegetation system, which is transitional between native and artificial vegetation systems. Modern pollen samples with HII values ≥ 38 are all distributed within long-established farmlands or in cities, completely controlled by human activity. Linear segment 3 represents artificial vegetation systems. Therefore, we identified an HII value of 22 ± 1.9 as the IP for distinguishing native (group 1) and nonnative vegetation systems (groups 2 and 3) over eastern China.

**Fig. 3.**
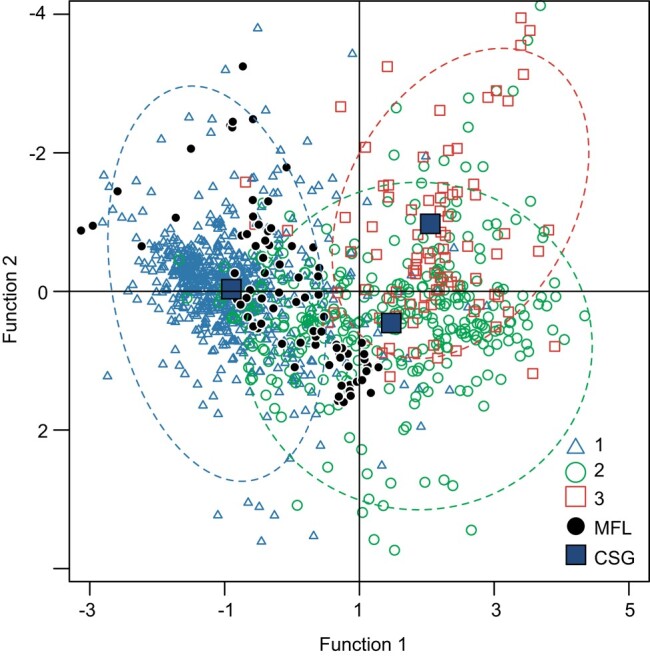
Results of discriminant analysis of modern pollen samples from eastern China and fossil pollen samples from the MFL profile at the Liangzhu site in the lower Yangtze River. 1, Modern pollen samples with HII values ≤ 22. 2, Modern pollen samples with HII values > 22 and < 38. 3, Modern pollen samples with HII values ≥ 38. MFL, Fossil pollen samples from the MFL profile of Liangzhu site. CSG, centers of sample groups.

To verify whether HII = 22 ± 1.9 and 38 ± 1.2 can serve as IPs for distinguishing native (group 1), secondary (group 2), and artificial vegetation systems (group 3), we conducted discriminant analysis of the modern pollen samples with these three a priori groups (Fig. 3). The cross-validation test of the discriminant functions (Table S1) showed that within the ranges of 800, 1,000, 1,200, and 1,400 km, the correct classification percentage for modern pollen samples in group 1 (corresponding to linear segment 1) is 90.2% on average. The correct classification percentages for groups 2 and 3 (corresponding to linear segments 2 and 3) are 58.4 and 44.8%, respectively (Table S1).

The results of discriminant analysis (Fig. 3) show that almost all fossil pollen samples from the MFL profile are assigned into groups 1 and 2, indicating that the HII = 22 effectively distinguishes native (group 1) and nonnative vegetation systems (groups 2 and 3), demonstrating the feasibility of the EIPDT in quantitatively distinguishing human impacts on paleovegetation.

### Applicability of the EIPDT in other regions of the world

**Fig. 4.**
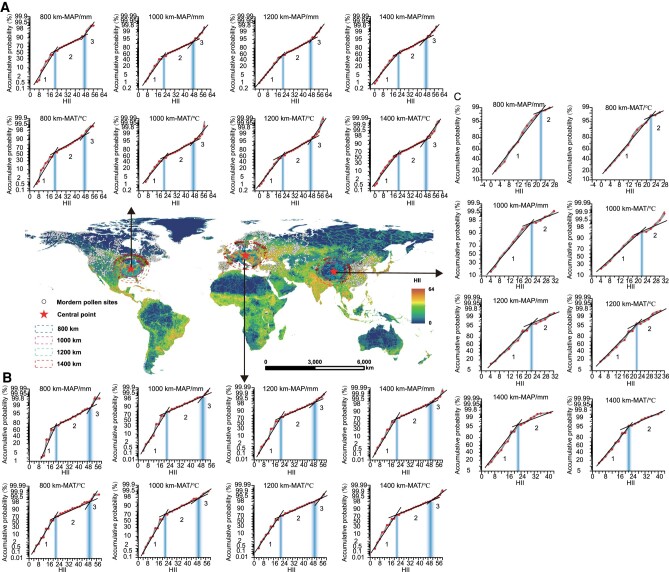
Distribution of IPs of vegetation systems in typical regions of the world. Global HII and cumulative probability curves of regression errors (WA-PLS, MAP/mm, and MAT/°C) as a function of HII for modern pollen samples from southeastern North America (A), Europe (B), and the Tibetan Plateau (C). Modern pollen samples are grouped by radii of 800, 1,000, 1,200, and 1,400 km. The gray shaded area represents the error range. The trend lines of the three scatter plots, characterized by different slopes, indicate distinct vegetation systems. The point of intersection between two linear segments marks the boundary between the two systems. The vertical axis uses a probability percentage scale. Probability percentage coordinates are nonequidistant coordinates with 50% as the center of symmetry and are plotted assuming a normal distribution (see Methods, Data S2–S4).

To verify the applicability of the EIPDT in distinguishing native and nonnative vegetation systems in other regions of the world, we randomly selected three locations in Europe (centered at 14.41°E, 49.57°N), North America (centered at 86.71°W, 37.98°N), and East Asia (the Tibetan Plateau, centered at 92.37°E, 35.39°N) with different environments and elevations, and conducted the same analysis (SI Appendix, Text E). The results reveal two IPs for two of these regions (as in eastern China), and one IP in another region: HII = 22 ± 2.4 and 48 ± 2.7 (Fig. 4A) in North America, HII = 22 ± 1.9 and 50 ± 2.5 (Fig. 4B) in Europe, and HII = 22 ± 1.4 (Fig. 4C) in the Tibetan Plateau.

It is evident that the HII values at the IP of native/secondary vegetation systems in the four regions are the same (∼22). This suggests a globally consistent pattern in changes of vegetation systems, i.e. the effects of human activities on native vegetation systems emerge when HII values reach or exceed 22. However, the HII values at the IPs of secondary/artificial vegetation systems in the four regions are different (Figs. 2 and 4): 50 ± 2.5 in Europe, 48 ± 2.7 in eastern North America, 38 ± 1.2 in eastern China, and absent in the Tibetan Plateau. This finding reveals regional differences in vegetation resilience to human disturbance and in human impact intensities due to differences in human population density, history, and management practices (48, 49). In Europe and North America, the conservation and management of vegetation began earlier, leading to large HII values at the IPs of secondary/artificial vegetation systems. In eastern China, the long history of human activities and the late implementation of vegetation conservation policies have resulted in serious damages to the secondary vegetation, leading to a low HII value (49, 50). In the Tibetan Plateau, the least anthropogenically disturbed region of China, it is not unexpected that no artificial vegetation system exists there.

Overall, the EIPDT provides the HII threshold value of native and nonnative vegetation systems worldwide, and thus it quantitatively distinguishes between anthropogenic and natural effects on vegetation. It also provides a foundation for reconstructing the evolution of native and nonnative paleovegetation, and for quantitatively reconstructing paleoclimate during periods when native vegetation prevailed.

### A case study: the application of the EIPDT into a fossil pollen record from the lower Yangtze River in China

#### Identification of fossil pollen spectra affected by human activities

**Fig. 5.**
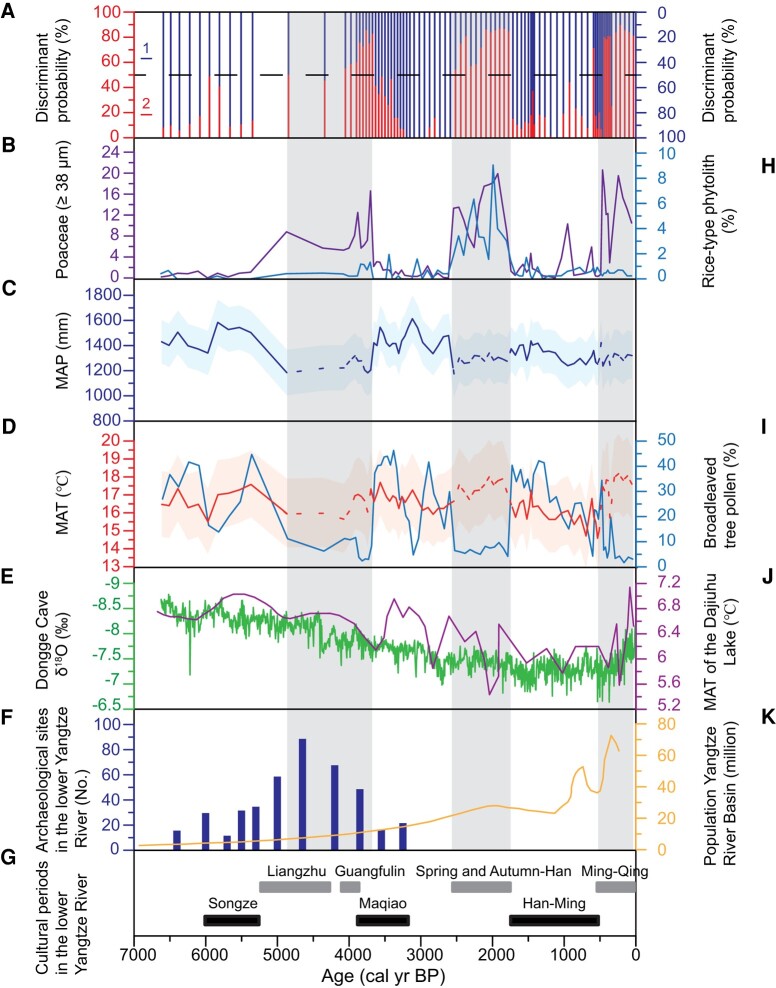
Environmental change in the Liangzhu area of the lower Yangtze River and comparison with other proxy records of climate and human activities in the East Asian monsoon region. A) Discriminant probability (1: native vegetation systems; 2: nonnative vegetation systems. The dotted line represents a 50% probability threshold, above which the sample probability falls within either group 1 or group 2) (this study). B) Percentages of Poaceae pollen (≥38 μm) (this study). C) Reconstructed MAP/mm (this study). D) Reconstructed MAT/°C (this study). E) δ^18^O record from Dongge Cave (51). F) Number of Neolithic archaeological sites in the lower Yangtze River (52). G) Cultural phases of the lower Yangtze River (46). H) Percentages of rice-type phytolith (47). I) Percentages of broadleaved tree pollen (this study). J) Pollen-based reconstruction of MAT/°C in the catchment of Dajiuhu Lake (11). K) Human population in the lower Yangtze River region (53). The shaded areas represent the range of error (C, D). The dotted lines indicate reconstructed MAP/mm and MAT/°C but unreliable due to nonnative vegetation (C, D).

Using the previously established discriminant functions of the three pollen groups defined by the two IPs at HII values of 22 and 38, we applied them into fossil pollen spectra from the MFL profile of the Liangzhu site to distinguish whether the spectra were from native (group 1) or nonnative vegetation systems (groups 2 and 3). The results (Fig. 5A) show that the samples of 5,000–3,700, 2,600–1,800, and 500–0 cal yr BP are assigned into group 2, implying that these three phases witnessed human-affected nonnative vegetation. The samples of 6,600–5,000, 3,700–2,600, and 1,800–500 cal yr BP are mainly assigned into group 1, suggesting that they are mainly from native vegetation, controlled by natural hydrothermal conditions, and thus that fossil pollen spectra of these three phases can be used for the quantitative reconstruction of past temperature and precipitation.

#### Relationship between the intensity of human activity and vegetation evolution in the Liangzhu region

##### Phases of enhanced human influence

The fossil pollen spectra during these phases (5,000–3,700, 2,600–1,800, and 500–0 cal yr BP) show the same pattern (Fig. S6), characterized by increased percentages of pioneer plants (e.g. *Pinus*), decreased percentages of zonal vegetation components such as *Liquidambar* and *Quercus* (including deciduous and evergreen) (Fig. 5I), and significant increases in cereal Poaceae (≥38 μm) and weed Poaceae (<38 μm), with an average of pollen percentage > 30%, suggesting that they were from nonnative vegetation significantly affected by human activities, as indicated by the EIPDT (Fig. 5B; SI Appendix, Text D).

These inferences are supported by regional archaeological evidence and other independent paleoenvironmental records (Fig. 5F and G) (12, 46). Phytolith analysis of the profile showed that a significant expansion of rice agriculture occurred after ∼5,000 cal yr BP, which was enabled by the development of advanced water management systems and tools such as plows and implements for harvesting rice plants (Fig. 5H) (47). Similar evidence was also found in fossil pollen and phytolith records of other sediment profiles at the Liangzhu and Qujialing sites (13, 54, 55). Furthermore, the phase of intensified human activity from 5,000 to 3,700 years ago corresponds to a period when there was an increase in the number of archaeological sites (Fig. 5F) (13, 52). The substantial population growth in the lower Yangtze River region during the phase of 2,600–1,800 cal yr BP, with the establishment of the Han Dynasty (202 BC) (Fig. 5K), is further evidence of the intensification of human activity (28, 53). Additionally, with the onset of the Iron Age, iron tools were introduced and extensively utilized in agriculture, which further enhanced the agricultural productivity (12, 56, 57). Therefore, human activities during these periods had significant impacts on the vegetation system in the lower Yangtze River (28, 55).

##### Phases of weakened human influence

Three phases of relatively weak human activity occurred in the Liangzhu region during 6,600–5,000, 3,700–2,600, and 1,800–500 cal yr BP. The pollen spectra of these phases share several common characteristics (Fig. S6): decreases in pioneer plants; significant increases in regional natural vegetation components such as *Liquidambar*, deciduous and evergreen *Quercus*; rare rice-type Poaceae pollen (≥38 μm) and the low abundance of weed Poaceae pollen (<38 μm) (<10%), supporting the results of the EIPDT (Fig. 5A).

The phases of weakened human influence on the native vegetation during 6,600–5,000 and 3,700–2,600 cal yr BP correspond approximately to two cultural phases: the Songze culture (5,900–5,300 cal yr BP) and the Maqiao culture (3,900–3,200 cal yr BP). These two cultures are characterized by a lower level of human activity compared to the Liangzhu culture (58), as indicated by low concentrations of rice phytoliths (Fig. 5H) (47). Furthermore, an ∼80% decrease in the number of archaeological sites during the phases before and after the Liangzhu culture indicate a socioeconomic decline (52, 59). During 1,800–500 cal yr BP, despite the increasing influence of human activities on vegetation during the Tang and Song dynasties, the vegetation in Liangzhu region was still dominated by native species (Fig. 5A), as it was not a socioeconomic and cultural center at that time (60). It is evident that archaeological evidence corroborates the EIPDT evidence for decreased human activity in the Liangzhu region during these three phases (13, 52).

To sum up, we have shown that the EIPDT effectively distinguished anthropogenic and natural influences on fossil pollen spectra, and that it provides a quantitative measure of the degree of human influence, which was not achieved in previous studies.

#### Quantitative reconstructions of temperature and precipitation using modern pollen training sets from native vegetation

According to the IPs of the EIPDT results, we developed a set of pollen–precipitation/temperature transfer functions using modern pollen training sets with HII ≤ 38 and HII ≤ 22 within 800, 1,000, 1,200, and 1,400 km of Liangzhu. We compared the lowest root mean square error of prediction (RMSEP) and the highest correlation coefficient (*R*^2^) (Table S2) of different transfer functions to determine the most reliable functions. The transfer functions established from modern pollen training sets with HII ≤ 22 have lower RMSEP values and higher *R*^2^ values, demonstrating that pollen–climate transfer functions developed using modern pollen training sets taken from native vegetation are superior (Fig. S8). We then applied them to the fossil pollen record from the Liangzhu to quantitatively reconstruct the paleoclimate.

The reconstructed mean annual precipitation (MAP/mm) and mean annual temperature (MAT/°C) during these three periods (6,600–5,000, 3,700–2,600, and 1,800–500 cal yr BP) over the last 6,600 years in the Liangzhu region show that MAP/mm fluctuated between 1,600 and 1,300 mm, and MAT/°C between 17.5 and 15°C (Fig. 5C and D). Both MAP/mm and MAT/°C exhibit a long-term decrease trend, similar to pollen-based paleoclimate records in the middle and lower Yangtze River (61). Due to the significant impact of human activities on vegetation during the other three periods (5,000–3,700, 2,600–1,800, and 500–0 cal yr BP), the reconstructed MAP/mm and MAT/°C are unreliable (dotted lines in Fig. 5C and D).

Our results are supported by other evidence from the East Asian monsoon region (11, 51, 62). For example, a pollen-based climate reconstruction from the Dajiuhu Lake catchment in the middle Yangtze River region (Fig. 5J) shows fluctuating decreases in precipitation and temperature over the past 6,600 years, attributed primarily to the weakening of the East Asian monsoon (11, 24), as suggested by δ^18^O records from Hulu Cave in Nanjing, Heshang Cave in Hubei, and Dongge Cave in Guizhou (Fig. 5E) (51, 62, 63).

In summary, we have developed a new approach, the EIPDT, for quantitatively differentiating pollen spectra from native and nonnative vegetation, which is replicable and reliable. Our approach addresses the longstanding problem in paleoecology of how to quantitatively distinguish between anthropogenic and natural effects on vegetation. Moreover, it can be applied beyond Quaternary pollen analysis, and it has potential applications in the studies of paleoecological signals potentially influenced by human activity.

## Methods

### Modern pollen database source

A total of 6,053 modern pollen samples from East Asia were used in this study, including 5,811 surface soil and moss samples and 242 lake surface samples (Fig. 1). Among them, 4,568 samples are from the East Asian modern pollen database (40, 64), and the other samples are from our research team (65) (SI Appendix, Text F). MAP and MAT data corresponding to modern pollen samples were extracted from the WorldClim dataset with a spatial resolution of ∼1 km^2^ (1,970–2,000) (https://www.worldclim.org/) (66). Additionally, the HII for modern pollen samples was derived from the global HII dataset with a spatial resolution of 1 km^2^ (1,995–2,004) (https://sedac.ciesin.columbia.edu/). This index integrates factors such as population density, land use/cover, infrastructure, and human activity characteristics including roads, railways, and navigable rivers. The HII ranges from 0 to 64 (41, 67).

The following procedures were used to obtain climate factors (MAP/mm and MAT/°C) and HII corresponding to the modern pollen data sample points: (i) The modern pollen data points were projected onto the WorldClim data and global HII raster datasets based on their latitude and longitude. (ii) MAP/mm, MAT/°C, and HII values for each modern pollen sample point were extracted by averaging their values within a 1 km² grid.

### Establishment of the pollen–climate transfer function method

Pollen–climate (MAP/mm, MAT/°C) transfer functions were established from the modern pollen samples within four different distances from the central point (800, 1,000, 1,200, and 1,400 km). Weighted averaging partial least sequence (WA-PLS) and the modern analog technique (68, 69) were used, which are the most commonly used methods in Holocene quantitative climate reconstructions because of their superior fitting performance and high predictive reliability (70). The predictive performances of all calibration models were assessed via self-cross-validation, and the performance statistics of each calibration model, including the RMSEP and *R*^2^ between observed and predicted values.

### Error inflection point-discriminant technique

**Fig. 6.**
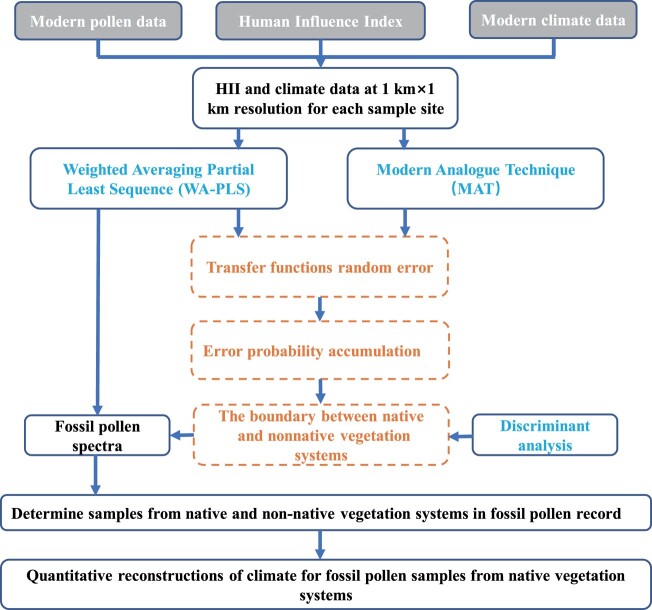
Flowchart of the application of the EIPDT.

Cao et al. (70) determined that the optimal range of modern pollen data for the quantitative reconstruction of regional climate using transfer functions was 800–1,500 km around fossil pollen sites. Hence, we concentrated on identifying the IP between native and nonnative vegetation systems within this range, at intervals of 200 km from the Liangzhu site. Details of the procedure are illustrated in Fig. 6.

Theoretically, vegetation can be classified into native and nonnative types in terms of the extent of natural and anthropogenic effects on vegetation (38). Native vegetation encompasses pristine vegetation and potential vegetation in a region, controlled by hydrothermal gradients (2, 17). Nonnative vegetation consists of secondary and artificial vegetation (17, 27). The artificial vegetation includes cultivated and anthropogenic vegetation such as agricultural fields, gardens, grasslands, anthropogenic forests, and urban green spaces (27). The composition and structure of artificial vegetation is often homogeneous due to long-term human cultivation practices. Nonnative vegetation, especially artificial vegetation, is mainly affected by human activities, but it is also partially affected by natural hydrothermal conditions. Therefore, the pollen–climate relationships for nonnative vegetation may be distorted and/or insignificant (5, 23).

Due to the different factors influencing native and nonnative vegetation, the regression errors in the transfer functions should be attributed to two distinct systems (43, 44). The random errors in each system follow a normal distribution, and the upper portion of the cumulative probability curve for each normal distribution corresponds to a linear segment (45). For a continuous random variable *X*, the cumulative distribution function *F*(*x*) is defined as


F(x)=P(X≤x)

where *X* is the random variable, *x* is the independent variable (−∞ < *x* < +∞), and *P* is the probability.

The differences in the length proportion, slope angle, and combination characteristics of the linear segments on the cumulative probability curve of the regression errors in the transfer functions can be used to determine and differentiate each system (45). Data processing was performed in C2 and Grapher 9 software.

Discriminant analysis is a mathematical technique for data classification, in which classification rules are derived by analyzing training sets and applied to classify unknown objects (71). In this study, discriminant functions were developed in terms of three a priori groups defined by the IPs of native/secondary and secondary/artificial vegetation systems, and used to verify the accuracy of the classification rules and identify modern analogs for fossil pollen spectra. The interpretation of discriminant analysis results follows the procedures of Liu and Lam (71). This analysis was conducted using SPSS 26.

## Supplementary Material

pgae135_Supplementary_Data

## Data Availability

All study data are included in the article and Supplementary material.
