# Supplementary material for: A novel approach for quantitatively distinguishing between anthropogenic and natural effects on paleovegetation
Source: PNAS Nexus. 2024 Mar 29;3(4):pgae135. doi: 10.1093/pnasnexus/pgae135 (PMC11010655; doi:10.1093/pnasnexus/pgae135)
Supplement: pgae135_Supplementary_Data [file pgae135_supplementary_data.zip › PNASNEXUS-PNASNEXUS-2024-00017R-s01.doc]

**Supplementary information for**

**A** **novel approach for** **quantitatively distinguishing between anthropogenic and** **natural effects on paleovegetation**

Baoshuo Fan^a,b^, Houyuan Lu^b,c^*, Yuecong Li^a^, Caiming Shen^d^*, Qinghai Xu^a^, Jianping Zhang^b^, Xiujia Huan^b,e^, Yonglei Wang^f^, Ningyuan Wang^f^, Deke Xu^b^, Yajie Dong^b^, Anning Cui^b^, Naiqin Wu^b^

^a^ Hebei Key Laboratory of Environmental Change and Ecological Construction, College of Geographical Sciences, Hebei Normal University, Shijiazhuang 050024, PR China; ^b^ Key Laboratory of Cenozoic Geology and Environment, Institute of Geology and Geophysics, Chinese Academy of Sciences, Beijing 100029, PR China; ^c^ University of Chinese Academy of Sciences, Beijing 100049, PR China; ^d^ Yunnan Key Laboratory of Plateau Geographical Processes and Environmental Changes, College of Tourism and Geographical Sciences, Yunnan Normal University, Kunming 650500, PR China; ^e^ Shandong Provincial Key Laboratory of Soil Conservation and Environmental Protection, School of Resources and Environment, Linyi University, Linyi 276000, PR China; ^f^ Zhejiang Provincial Institute of Relics and Archaeology, Hangzhou 310014, PR China

* Corresponding author: Houyuan Lu, Caiming Shen

E-mail: [houyuanlu@mail.iggcas.ac.cn](mailto:houyuanlu@mail.iggcas.ac.cn); [cmshen@hotmail.com](mailto:cmshen@hotmail.com)

**This PDF file includes:**

Supplementary text

Figures S1 to S8

Tables S1 to S2

SI References

**Supplementary Information Text**

**A. Environmental background of the Liangzhu archaeological site area in China**

The Liangzhu archaeological site is located in the northern part of Zhejiang Province, China, at the boundary between the western Tianmu Mountain and the Hangjiahu Plain. The eastern and western sides of the sites are connected to the Hangjiahu Plain and the hilly mountainous area of the Tianmu Mountain range (1). The northern, western, and southern sides of the study area consist of low hills, forming a 'C'–shaped topography with mountains encircling it on three sides. Within this 'C'–shaped topography, there are a few low–lying hills with elevations of tens of meters in the middle and eastern parts. The overall terrain of the Liangzhu ancient city area is relatively flat, with most areas ranging from 2 to 3 m above sea level (2) (*SI Appendix*, Fig. S1).

The study area has subtropical monsoon climate, with a mean annual temperature (MAT/℃) of around 15–16°C and a mean annual precipitation (MAP/mm) of approximately 1300 mm. Regional vegetation is characterized by zonal one, specifically subtropical evergreen and deciduous broadleaved mixed forest, comprising typical arboreal species such as *Cyclobalanopsis*, *Quercus*, *Liquidambar*, *Castanea*, *Cunninghamia*, *Celtis*, and *Pinus* (3). Additionally, *Bamboo* forests are widely distributed in the region, especially in the Tianmu Mountain area. Shrubbery is primarily found in the western and northern hilly areas (4).

**B. Cultural background of the Liangzhu archaeological site**

The Liangzhu culture (5300–4300 cal yr BP) is regarded as evidence of a five-thousand-year Chinese civilization. It is famous for its highly developed rice agriculture, large-scale hydraulic engineering, and exquisite jade artifacts, representing the pinnacle of the Neolithic era in the lower Yangtze region (1, 5). In terms of the number of Liangzhu cultural archaeological sites, there are more than 530 sites in the Hangjiahu Plain alone, making it the region with the highest number of sites among all Neolithic cultures in this area (6). The size and quantity of these sites far exceed those of the previous Songze culture (7).

In addition to the quantity and size of the archaeological sites, Liangzhu jade artifacts have also attracted scholars' attention (8). A large number of Jade Cong, Jade Bi, Jade Bracelets, Jade Yue, and Jade Huang have been unearthed from the Liangzhu site (*SI Appendix*, Fig. S2). The craftsmanship of the Liangzhu culture's jade artifacts has reached an extremely exquisite level, and various techniques are used to depict the patterns on the jade objects, including incision, relief, and hollow carving (8).

Furthermore, during the Liangzhu culture phases, the scale of rice fields expanded. Up to now, a single field with an area of approximately 1000 m^2^ has been discovered, with the largest reaching nearly 2000 m^2^ (9). In comparison, the maximum area of a single rice field excavated from the Majiabang culture is only around 16 m^2^, and for the Songze culture, it is around 100 m^2^ (10).

The Liangzhu ancient city possesses a complete structure from the inside out, consisting of the inner palace, royal city, outer enclosure, and peripheral water management system (11). The outer water management system not only represents the earliest large-scale hydraulic engineering project in China but also the world's earliest flood control dam system. This water management system is distributed in the northern and western parts of the ancient city, comprising of 11 dams, which can be divided into three sections: terraced embankments, high dams, and low dams. The construction volume amounts to 2.6×10^6^ m^3^ of earthwork, and it covers an area of over 100 km^2^ (12).

**C. Lithology and chronology of the MFL sedimentary profile at the Liangzhu site**

The MFL profile (119.983°E, 30.397°N, 30 m a.s.l.) was located within the MFL reservoir and has a total depth of 188 cm. It was divided into 7 layers from bottom to top (Fig. 1C): Layer 7 (188–185 cm) and Layer 6 (185–164 cm) consist of yellow-brown silt; Layer 5 (164–130 cm) consists of gray-yellow silt; Layer 4 (130–72 cm) consists of dark-gray clayey silt; Layer 3 (72–42 cm) is composed of light-gray silty clay, including some pottery shards; Layer 2 (42–30 cm) consists of gray-yellow silty deposits; Layer 1 (30–0 cm) is modern yellow topsoil. Sampling was conducted every 5 cm in Layer 1 and every 2 cm in Layers 2–7, resulting in a total of 85 samples.

The chronology of the MFL profile includes 12 dating samples, comprising 3 OSL dating and 9 AMS^14^C dating (two charcoal, one plant remains, and six organic sediments). Age–depth models were established using the Bacon function in the R programming language (13) (*SI Appendix*, Fig. S3). The chronological range of the MFL profile spans 6600–0 cal yr BP (14).

**D. Pollen analysis and dominant pollen types of the MFL profile from the Liangzhu site**

Pollen analysis was conducted following the conventional HCl-NaOH-HF method for sample preparation (15). Each sample, weighing 2 g prior to chemical treatment, was supplemented with one *Lycopodium* spores (27,560 spores) to calculate pollen concentrations. Pollen identification and quantification were performed under a 400x Carl Zeiss (ZEISS) optical microscope, and more than 500 terrestrial pollen grains were identified and counted for each sample. The primary references for pollen identification were "Flora Pollen Morphology in China" (16), and "Pollen Atlas of Quaternary China" (17). To differentiate between pollen grains from the Poaceae family associated with rice cultivation and those from non-crop Poaceae, prior research findings on pollen grain size criteria were employed (18). Poaceae pollen grains were divided into two groups based on size, one group with grain size ≥38 μm representing rice cultivation, the other with grain size <38 μm representing non-crop Poaceae. Pollen spectra were calculated based on the total count of identified terrestrial pollen grains. Tilia software was employed to plot pollen diagrams, and pollen spectra were subjected to ordination and cluster analysis using the CONISS technique for pollen zone subdivision. Sample preparation and pollen identification were conducted at the Key Laboratory of Cenozoic Geology and Environment, Institute of Geology and Geophysics, Chinese Academy of Sciences and the College of Geographical Sciences, Hebei Normal University.

A total of 104 pollen and spore taxa were identified from 85 samples in the MFL profile of the Liangzhu site, including 26 taxa of arboreal pollen, 10 taxa of shrubs pollen, 51 taxa of herbaceous pollen, and 17 taxa of ferns spores, with a total count of 48,892 pollen grains identified. Common arboreal pollen taxa include *Pinus*, evergreen *Quercus*, deciduous *Quercus*, *Pterocarya*, *Liquidambar*, *Ulmus*, *Alnus*, *Betula*, *Tilia*, etc. Rosaceae is common shrub pollen. Common terrestrial herbaceous pollen taxa include weed Poaceae <38 μm, cereal Poaceae ≥38 μm, Asteraceae, Cyperaceae, Brassicaceae, Polygonaceae, Caryophyllaceae, Rhamnaceae, Valerianaceae, etc. Common aquatic pollen taxa include *Myriophyllum*, *Nymphoides*, *Typha*, and *Trapa*. Common fern spores include Monoletes and Triletes **(***SI Appendix*, Fig. S4 and Fig. S5**)**. According to the CONISS cluster analysis results of pollen spectra from the MFL profile, fossil pollen spectra of the MFL profile can be divided into 6 pollen zones (*SI Appendix*, Fig. S6).

Zone 1 (188–168 cm; 6600–5000 cal yr BP): This zone is dominated by *Pinus* and *Liquidambar* pollen, accounting for ca. 60% of the total pollen. Herbaceous pollen percentages are the lowest throughout the profile, averaging 20.4%, primarily composed of weed Poaceae (<38 μm) at 10.8%, Cyperaceae at 5%, and rare cereal Poaceae (≥38 μm) at 0.7%.

Zone 2 (168–148 cm; 5000–3700 cal yr BP): This zone is characterized by significant decreases (ca. 15%) in pollen percentages of broadleaf trees (such as *Liquidambar*, deciduous *Quercus*, and evergreen *Quercus*) and marked increases of Poacece pollen (both <38 μm and ≥38 μm, together with an increase by ca. 30%). In this zone, *Pinus* and Cyperaceae pollen increase to averages of 33% and 4%.

Zone 3 (148–108 cm; 3700–2600 cal yr BP): This zone is marked by ca. 15% increases in the pollen percentages of broadleaf trees (including deciduous *Quercus*, evergreen *Quercus*, and *Liquidambar*) and ca. 25% decreases in *Pinus* pollen percentages. Poaceae pollen (both <38 μm and ≥38 μm) percentages decrease by ca. 20%, and Cyperaceae pollen percentages increase by ca. 10%. The spores of Polypodiaceae became significantly abundant at 3300 cal yr BP, with an increase of ca. 40%.

Zone 4 (108–84 cm; 2600–1800 cal yr BP): This zone is characterized by ca. 30% and 20% increases of *Pinus* pollen and Poaceae pollen (both <38 and ≥38 μm) at the expense of broadleaf tree pollen such as deciduous *Quercus*, evergreen *Quercus*, and *Liquidambar*, as well as Cyperaceae pollen (decreases by ca. 16%). The spores of Polypodiaceae decrease to an average of 0.8%.

Zone 5 (84–38 cm; 1800–500 cal yr BP): This zone is marked by ca. 20% increases in the pollen percentages of broadleaf trees such as deciduous *Quercus*, evergreen *Quercus*, and *Liquidambar* as well as ca. 30% decreases in *Pinus* pollen percentages again. Cyperaceae pollen percentages increase by ca. 35% at the expense of Poaceae pollen (both <38 and ≥38 μm) percentages. The spores of Polypodiaceae slightly increase to ca. 5%.

Zone 6 (38–0 cm; 500–0 cal yr BP): This zone is distinguished from zone 5 by decreases in pollen percentages of broadleaf trees (deciduous *Quercus*, evergreen *Quercus*, and *Liquidambar*) and Cyperaceae, and increases in *Pinus* pollen (ca. 20%) and Poaceae pollen (both <38 and ≥38 μm) (ca. 30%). The spores of Polypodiaceae also decrease further to below 1%.

**E. Sources of modern pollen data from Eurasian and North America region**

Modern pollen data in Eurasian were sourced from Eurasian Modern Pollen Database (19), and it encompasses a total of 8,747 samples. Among them, 2,480 are lake surface samples and peat samples, 3,474 are moss samples, 1,307 are topsoil samples, and 1,486 are from unknown sampling environments.

For North America, modern pollen data were obtained from Pollen Database of North American and Greenland (20) comprising 4,634 samples. Out of these, 2,261 are lake surface samples, 338 are peat samples, 860 are moss samples, 556 are topsoil samples, and 580 samples are from unknown sampling environments.

To verify the applicability of the EIPDT in distinguishing native and non-native vegetation systems in other regions of the world, we also randomly selected three locations in Europe, North America, and East Asia to do the same work as above. Europe and North America represent regions with relatively enhanced human activities, while the Tibetan Plateau of China represents a region with relatively weakened human activity. The central coordinates for each region were as follows: Europe (14.41°E, 49.57°N), North America (86.71°W, 37.98°N), and the Tibetan Plateau of China (92.37°E, 35.39°N). Modern pollen samples within four distance ranges of 800, 1000, 1200, and 1400 km were collected around each central point to construct a new modern pollen database.

**F. Screening of modern pollen databases**

The screening of modern pollen data is crucial for ensuring the accuracy of quantitative reconstructions. Thus, we conducted a selection process for the modern pollen database based on the following criteria: 1) Air traps and dust samples were excluded from modern pollen samples due to differences in the preservation of modern pollen compared to fossil pollen. 2) Remove samples whose latitude, longitude, and altitude do not match the real situation; a total of 18 samples were removed in this study. 3) Considering the influence of human activities in certain areas over the past two decades, samples, whose Human Influence Index (HII) values for 1995–2004 were inconsistent with their original sampling records, were excluded; a total of 98 samples were excluded in this study.

Geographical distance selection is one important step in these most commonly used methods because the training set composed of modern pollen within appropriate spatial ranges is crucial for the model selection. If the range of the training set is too small, it may not fully cover the climatic variations in the reconstruction sequence. Conversely, if the coverage range is too large, the pollen-climate relationship may be weakened, leading to significant errors (19, 20). In this study, we analyzed the modern pollen data from four different radii of 800, 1000, 1200, and 1400 km (*SI Appendix*, Fig. S7).

**Figures S1 to S8**

**Fig. S1.** **The structure of Liangzhu hydraulic system and the geographical location of the MFL profiles at the Liangzhu site.**

**Fig. S2.** **Representative cultural relics and artifacts from the Liangzhu culture layer**. **(*A*)** Jade Cong. (***B*)** Jade Bi. (***C*)** Jade Axe. (***D*)** Jade Bracelet. (***E*)** Pottery Tripod Cauldron. (***F*)** Pottery Jar (21).

**Fig. S3. The age-depth model of the MFL profile at the Liangzhu site** (adapted from Huan et al. (14)).

**Fig. S4. The common pollen taxa in the MFL profile at the Liangzhu site.** **(*A*)** *Piuns*. **(*B*)** deciduous *Quercus*. (***C, D*)** evergreen *Quercus*. (***E, F*)** *Liquidambar*. (***G*)** *Fagus*. (***H*)** *Ulmus*. **(*I*)** *Celtis*. (***J*)** *Pterocarya*. (***K*)** *Juglans*. (***L*)** *Alnus*. (***M*)** *Tilia*. (***N*)** *Betula*. (***O*)** Poaceae (< 38 μm). (***P, Q*)** Poaceae (≥38 μm). (***R*)** Chenopodiaceae. **(*S*)** *Artemisia*. (***T*)** Brassicaceae. (***U*)** Caryophyllaceae. (***V*)** *Thalictrum*.

**Fig. S5. The common pollen taxa in the MFL profile at the Liangzhu site.** (***A*)** Polygonaceae. (***B*)** *Xanthium*. (***C*)** Asteraceae. (***D*)** *Taraxacum*. (***E*)** Cyperaceae. (***F*)** Rhamnaceae. (***G*)** Valerianaceae. (***H*)** *Myriophyllum*. (***I*)** *Typha*. (***J*)** *Nymphoides*. (***K*)** *Trapa*. (***L*)** Gleicheniaceae. (***M*)** Sinopteridaceae. (***N*)** *Osmunda*. (***O*)** Pteridaceae. (***P*)** Monolete.

**Fig. S6. Pollen percentage diagram of main pollen taxa from the MFL profile at the Liangzhu site.**

**Fig. S7.** **MAP/mm and MAT/℃ frequency distributions of modern pollen samples (HII value less than 22) within 1200 km. (*A*)** MAT/℃. (***B*)** MAP/mm.

**Fig. S8. Scatter plots of estimated versus observed values and residuals versus observed values for pollen-MAP/mm (*A*) and pollen-MAT/℃ (*B*) transfer functions developed by WA-PLS** **using modern pollen training-set from native vegetation** **within 1200 km.**

**Tables S1 and S2**

**Table S1.** **The discriminant analysis results of modern pollen samples are presented in terms of their relationships with the HII.** (within the range of 800, 1000, 1200, and 1400 km centered around the Liangzhu site)

| Distance | N |  |  | HII | 1 | 2 | 3 | Percentage correctly  classified |
| --- | --- | --- | --- | --- | --- | --- | --- | --- |
| 800 km | 245 | Original | Count | 1 | 155 | 3 | 1 | 93.5% |
|  |  |  |  | 2 | 9 | 70 | 0 |  |
|  |  |  |  | 3 | 0 | 3 | 4 |  |
|  |  |  | % | 1 | 97.5 | 1.9 | 0.6 |  |
|  |  |  |  | 2 | 11.4 | 88.6 | 0 |  |
|  |  |  |  | 3 | 0 | 42.9 | 57.1 |  |
|  |  | Cross-validation | Count | 1 | 140 | 14 | 5 | 80.4% |
|  |  |  |  | 2 | 21 | 55 | 3 |  |
|  |  |  |  | 3 | 1 | 4 | 2 |  |
|  |  |  | % | 1 | 88.1 | 8.8 | 3.1 |  |
|  |  |  |  | 2 | 26.6 | 69.6 | 3.8 |  |
|  |  |  |  | 3 | 14.3 | 57.1 | 28.6 |  |
| 1000 km | 681 | Original | Count | 1 | 363 | 13 | 7 | 90.9% |
|  |  |  |  | 2 | 26 | 162 | 40 |  |
|  |  |  |  | 3 | 0 | 22 | 48 |  |
|  |  |  | % | 1 | 94.8 | 3.4 | 1.8 |  |
|  |  |  |  | 2 | 11.4 | 71.1 | 17.5 |  |
|  |  |  |  | 3 | 0 | 31.4 | 68.6 |  |
|  |  | Cross-validation | Count | 1 | 355 | 21 | 7 | 89.9% |
|  |  |  |  | 2 | 38 | 143 | 47 |  |
|  |  |  |  | 3 | 1 | 28 | 41 |  |
|  |  |  | % | 1 | 92.7 | 5.5 | 1.8 |  |
|  |  |  |  | 2 | 16.7 | 62.7 | 20.6 |  |
|  |  |  |  | 3 | 1.4 | 40 | 58.6 |  |
| 1200 km | 1155 | Original | Count | 1 | 667 | 50 | 10 | 79.8% |
|  |  |  |  | 2 | 71 | 206 | 59 |  |
|  |  |  |  | 3 | 5 | 38 | 48 |  |
|  |  |  | % | 1 | 91.7 | 6.9 | 1.4 |  |
|  |  |  |  | 2 | 21.1 | 63.3 | 17.6 |  |
|  |  |  |  | 3 | 5.5 | 41.8 | 52.7 |  |
|  |  | Cross-validation | Count | 1 | 661 | 52 | 14 | 76.6% |
|  |  |  |  | 2 | 81 | 189 | 66 |  |
|  |  |  |  | 3 | 9 | 48 | 34 |  |
|  |  |  | % | 1 | 90.9 | 7.2 | 1.9 |  |
|  |  |  |  | 2 | 24.1 | 56.3 | 19.6 |  |
|  |  |  |  | 3 | 9.9 | 52.7 | 37.4 |  |
| 1400 km | 1777 | Original | Count | 1 | 1067 | 102 | 25 | 76.4% |
|  |  |  |  | 2 | 145 | 229 | 114 |  |
|  |  |  |  | 3 | 8 | 26 | 61 |  |
|  |  |  | % | 1 | 89.4 | 8.5 | 2.1 |  |
|  |  |  |  | 2 | 29.7 | 46.9 | 23.4 |  |
|  |  |  |  | 3 | 8.4 | 27.4 | 64.2 |  |
|  |  | Cross-validation | Count | 1 | 1065 | 102 | 27 | 75.2% |
|  |  |  |  | 2 | 147 | 219 | 122 |  |
|  |  |  |  | 3 | 9 | 34 | 52 |  |
|  |  |  | % | 1 | 89.2 | 8.5 | 2.3 |  |
|  |  |  |  | 2 | 30.1 | 44.9 | 25 |  |
|  |  |  |  | 3 | 9.5 | 35.8 | 54.7 |  |

Note: 1. Modern pollen samples with HII values ≤ 22. 2: Modern pollen samples with HII values values > 22 and < 38. 3: Modern pollen samples with HII values ≥ 38.

**Table S2.** **The outcomes of pollen-climate transfer functions established by different methods and training-sets.** (samples with HII values below 38 and 22)

| Factor | HII | Transfer functions | Distance (km) | N | RMSE | R^2^ | RMSEP | Boot_R_2_ | t-test |
| --- | --- | --- | --- | --- | --- | --- | --- | --- | --- |
| MAP/mm | HII≤22 | WA-PLS | 800 | 159 | 124.92 | 0.8854 | 198.36 | 0.7486 | 0.002 |
|  |  |  | 1000 | 383 | 173.78 | 0.8615 | 214.04 | 0.8047 | 0.001 |
|  |  |  | **1200** | **727** | **170.96** | **0.8873** | **193.09** | **0.8577** | **0.001** |
|  |  |  | 1400 | 1194 | 182.47 | 0.8709 | 204.73 | 0.8445 | 0.001 |
|  |  | MAT | 800 | 159 | 181.22 | 0.7746 | 189.24 | 0.7837 |  |
|  |  |  | 1000 | 383 | 182.25 | 0.8554 | 193.67 | 0.8580 |  |
|  |  |  | 1200 | 727 | 188.03 | 0.8698 | 198.17 | 0.8716 |  |
|  |  |  | 1400 | 1194 | 193.22 | 0.8657 | 204.88 | 0.8655 |  |
| MAP/mm | HII≤38 | WA-PLS | 800 | 238 | 166.84 | 0.8241 | 224.25 | 0.7088 | 0.002 |
|  |  |  | 1000 | 611 | 183.78 | 0.8647 | 216.80 | 0.8245 | 0.001 |
|  |  |  | 1200 | 1063 | 181.82 | 0.8659 | 204.90 | 0.8388 | 0.001 |
|  |  |  | 1400 | 1682 | 189.20 | 0.8486 | 208.93 | 0.8227 | 0.001 |
|  |  | MAT | 800 | 238 | 189.94 | 0.7798 | 199.74 | 0.7851 |  |
|  |  |  | 1000 | 611 | 198.03 | 0.8407 | 212.26 | 0.8442 |  |
|  |  |  | 1200 | 1063 | 191.37 | 0.8531 | 204.86 | 0.8538 |  |
|  |  |  | 1400 | 1682 | 200.38 | 0.8363 | 214.09 | 0.8307 |  |
| MAT/℃ | HII≤22 | WA-PLS | 800 | 159 | 1.6211 | 0.7179 | 2.3071 | 0.4877 | 0.01 |
|  |  |  | 1000 | 383 | 1.9570 | 0.8397 | 2.4603 | 0.7694 | 0.001 |
|  |  |  | **1200** | **727** | **2.2548** | **0.8351** | **2.4992** | **0.7974** | **0.001** |
|  |  |  | 1400 | 1194 | 2.4826 | 0.7992 | 2.7854 | 0.7606 | 0.001 |
|  |  | MAT | 800 | 159 | 2.327 | 0.3885 | 2.5499 | 0.3823 |  |
|  |  |  | 1000 | 383 | 2.6339 | 0.7128 | 2.7533 | 0.7218 |  |
|  |  |  | 1200 | 727 | 2.5383 | 0.7932 | 2.6734 | 0.7959 |  |
|  |  |  | 1400 | 1194 | 2.6122 | 0.7820 | 2.7381 | 0.7843 |  |
| MAT/℃ | HII≤38 | WA-PLS | 800 | 238 | 1.5587 | 0.6923 | 2.1860 | 0.4661 | 0.021 |
|  |  |  | 1000 | 611 | 2.0529 | 0.7712 | 2.4038 | 0.7029 | 0.001 |
|  |  |  | 1200 | 1063 | 2.4171 | 0.7789 | 2.6217 | 0.7459 | 0.001 |
|  |  |  | 1400 | 1682 | 2.7018 | 0.7560 | 2.8891 | 0.7270 | 0.001 |
|  |  | MAT | 800 | 238 | 2.1850 | 0.3984 | 2.2947 | 0.4041 |  |
|  |  |  | 1000 | 611 | 2.2661 | 0.7266 | 2.3819 | 0.7317 |  |
|  |  |  | 1200 | 1063 | 2.3033 | 0.8016 | 2.4213 | 0.8054 |  |
|  |  |  | 1400 | 1682 | 2.4935 | 0.7955 | 2.6211 | 0.7973 |  |

**SI References**

1. Liu B, et al. 2017. Earliest hydraulic enterprise in China, 5,100 years ago. *Proc Natl Acad Sci U S A.* 114:13637–13642.
2. Liu B, Wang NY, Chen MH. 2019. From archaeological site to world cultural heritage: The value identification and protection of Liangzhu ancient city (in Chinese). *Southeast Culture*. 6–13.
3. Cai RH, He SJ. 1980. Vegetation types and their distribution in west lake mountain of Hangzhou city, China (in Chinese). *J Zhejiang Univ Sci Ed.* 4: 100–112.
4. Wu ZY. 2018. Chinese Vegetation (in Chinese). (Beijing: Science Press, 1980).
5. C. Renfrew, B. Liu, The emergence of complex society in China: the case of Liangzhu. *Antiquity* 92:975–990.
6. Liu Y, et al. 2014. Palaeoclimatic and environmental changes since the Mid–Holocene in the Hangzhou Bay and their possible impacts on the evolution of Liangzhu Culture (in Chinese). *J Lake Sci.* 26:322–330.
7. Ding P. 2000. The scope of Liangzhu ancient country and its main characteristics of cultural exchanges with neighboring countries (in Chinese). *Prehistory.* 1: 577–588.
8. Liu B. 2021. Liangzhu and centennial archaeology: The underestimated neolithic age in China (in Chinese). *Chinese Culture Research.* 114:1–11.
9. Guo LX, Guo JY. 2016. Types of early rice fieldremains and their social correlation (in Chinese). *Agricultural History of China.* 35:13–28.
10. Ding JL. 2004. The origin of paddy fields and rice–farming in the lower reaches of the Yangtze River in the Neolithic Age (in Chinese). *Southeast Culture.* 2: 19–23.
11. Wang NY, Liu B. 2015. The survey of the water engineering on the periphery of the Liangzhu Ancient City in Hangzhou (in Chinese). *Archaelogy.* 568:3–13.
12. Wang N. 2016. Survey and excavation of the water conservancy system in Liangzhu city and surroundings (in Chinese). *Research on Heritages and Preservation.* 1:102–110.
13. Blaauw M, Christen JA. 2011. Flexible paleoclimate age–depth models using an autoregressive gamma process. *Bayesian Analysis.* 6:457–474.
14. Huan XJ, et al. 2022. Intensification of rice farming and its environmental consequences recorded in a Liangzhu reservoir, China. *Quat Int.* 619:39–45.
15. Fægri K, et al. 1989. Textbook of pollen analysis. *J Biogeogr.* 12:328.
16. Wang FX, et al. 1995. Pollen Morphology in China. Beijing: Science Press. 1‒461.
17. Tang LY, et al. 2018. An Illustrated Handbook of Quaternary Pollen and Spores in China (in Chinese). (Science Press, Beijing).
18. Li CH, et al. 2012. Understanding the ecological background of rice agriculture on the Ningshao Plain during the Neolithic Age: pollen evidence from a buried paddy field at the Tianluoshan cultural site. *Quat Sci Rev.* 35:131–138.
19. Chevalier M, et al. 2019. Eurasian Modern Pollen Database (former European Modern Pollen Database), *Pangaea*. https://doi.pangaea.de/10.1594/PANGAEA.909130
20. Whitmore JK, et al. 2005. Modern pollen data from North American and Greenland for multi-scale paleoenvironmental applications. *Quat Sci Rev.* 24: 1828–1848.
21. Chen MH, et al. 2021. Brief report on excavation of Jiangjiashan Cemetery in Liangzhu Ancient City, Yuhang District, Hangzhou (in Chinese). *Archaelogy.* 645: 35–55.
